# Case report: Mutation evolution in a patient with TdT positive high grade B cell lymphoma with MYC and BCL2 rearrangements following the treatment of concurrent follicular lymphoma and diffuse large B-cell lymphoma

**DOI:** 10.1007/s12672-024-00991-5

**Published:** 2024-04-25

**Authors:** Fen Zhang, Yu Chen, Qian Cui, Yan Ge, Yanhui Liu

**Affiliations:** Department of Pathology, Guangdong Provincial People’s Hospital (Guangdong Academy of Medical Sciences), Southern Medical University, No. 106, 2nd Zhongshan Road, Guangzhou, 510080 China

**Keywords:** Follicular lymphoma, Diffuse large B-cell lymphoma, Terminal deoxynucleotydil transferase, Double hit high grade B cell lymphoma, Mutation landscape

## Abstract

**Background:**

Concurrent follicular lymphoma (FL) and diffuse large B-cell lymphoma (DLBCL)was reported in some studies, while the diagnosis of TdT (terminal deoxynucleotydil transferase) positive high grade B cell lymphoma (HGBL) with MYC and BCL2 rearrangements (“double hit”) transformed from FL/DLBCL has been rarely reported. Herein, we described the clinical features and mutation profiles of a case diagnosed with TdT positive “double hit” HGBL following the treatment of FL/DLBCL.

**Case presentation:**

This is a 43-year-old Chinese man who was diagnosed with low grade FL (account for 80%) combined with DLBCL (20%) at a stage of IVB. The patient presented with *BCL2*/*IGH* translocation without *MYC* rearrangement, as well as the expressions of CD20, CD19, CD10 and BCL2 at the initial diagnosis of FL/DLBCL. *MYC* rearrangement and TdT expression occurred after the treatment. The targeted sequencing revealed mutations in *KMT2D*, *FOXO1*, *CREBBP*, *ATM*, *STAT6*, *BCL7A*, *DDX3X*, *MUC4*, *FGFR3*, *ARID5B*, *DDX11* and *PRKCSH* genes were the co-mutations shared by the FL/DLBCL and TdT positive “double hit” HGBL, while *CCND3*, *BIRC6*, *ROBO1* and *CHEK2* mutations specifically occurred after the treatment. The overall survival time was 37.8 and 17.8 months after the initial diagnosis of FL/DLBCL and TdT positive “double hit” HGBL, respectively.

**Conclusion:**

This study reports a rare case of TdT positive “double hit” HGBL following the treatment of concurrent FL/DLBCL and highlights the mutation characteristics. Collectively, this study will help enrich the knowledge of TdT positive “double hit” HGBL transformed from FL/DLBCL.

**Supplementary Information:**

The online version contains supplementary material available at 10.1007/s12672-024-00991-5.

## Introduction

Follicular lymphoma (FL) is a malignant counterpart of normal germinal center B-cells, which is the second most common lymphoma behind diffuse large B cell lymphoma (DLBCL) [1]. Translocation of t(14;18) is a common event which is detected in about 85% FL cases, leading to the overexpression of BCL2, an anti-apoptotic protein. However, t(14;18) is neither required nor sufficient for the development of FL [2, 3]. The mutations of chromatin modifying-related genes (*KMT2D, CREBBP, EZH2*) are other common features of FL [4]. The prognosis of FL is good with a 10-year overall survival (OS) of approximately 80% due to the development of rituximab [5]. However, 2–3% of FL cases suffer from histological transformation into high-grade lymphoma every year [6], for whom the prognosis is poor with a median survival of around 3.8 years [7].

The most common histological transformation of FL is DLBCL, with a varied incidence from 30 to 60% [8]. Less than 10% of FL patients present with DLBCL (FL/DLBCL) concurrently at initial diagnosis, and the prognosis for them is poor with a median survival interval of 14–27 months [9, 10]. FL/DLBCL has been reported in some studies [11, 12], but to our knowledge, the diagnosis of TdT (terminal deoxynucleotydil transferase) positive “double hit” high grade B cell lymphoma (HGBL) following the treatment of FL/DLBCL has been rarely reported [13]. Herein, we described the clinical and mutation features of a case with TdT positive “double hit” HGBL following the treatment of FL/DLBCL.

## Case presentation

**Fig. 1:**
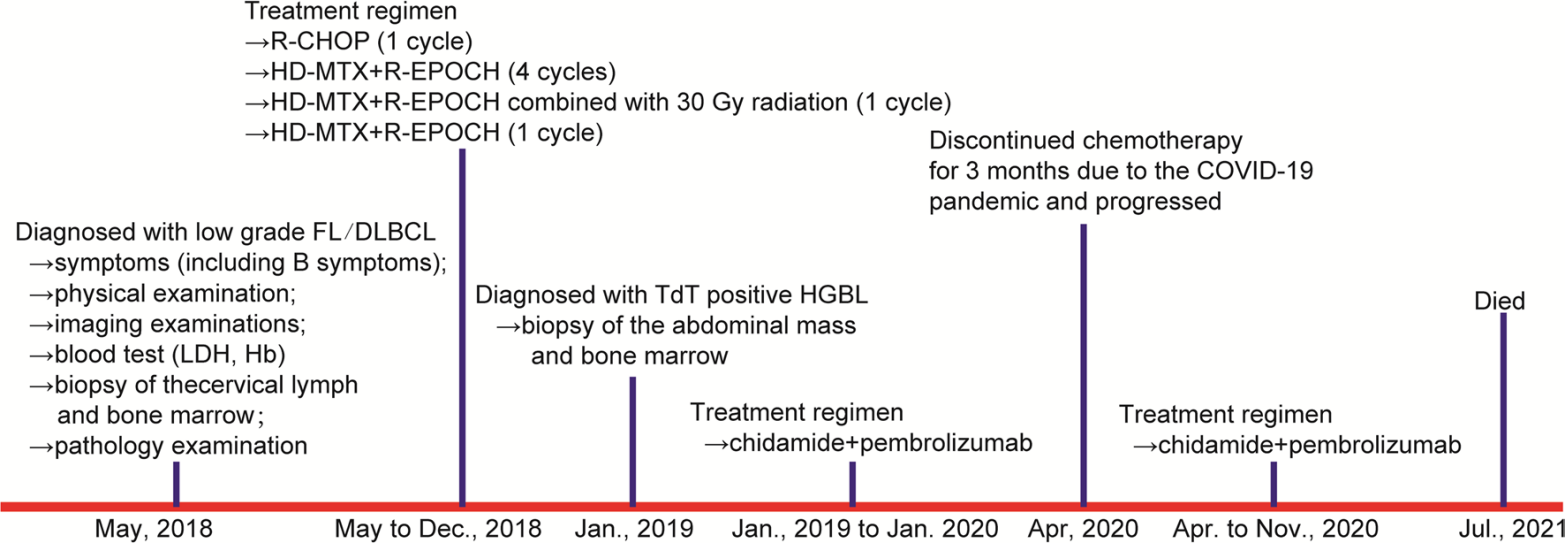
Diagnosis and treatment process of this patient

**Fig. 2:**
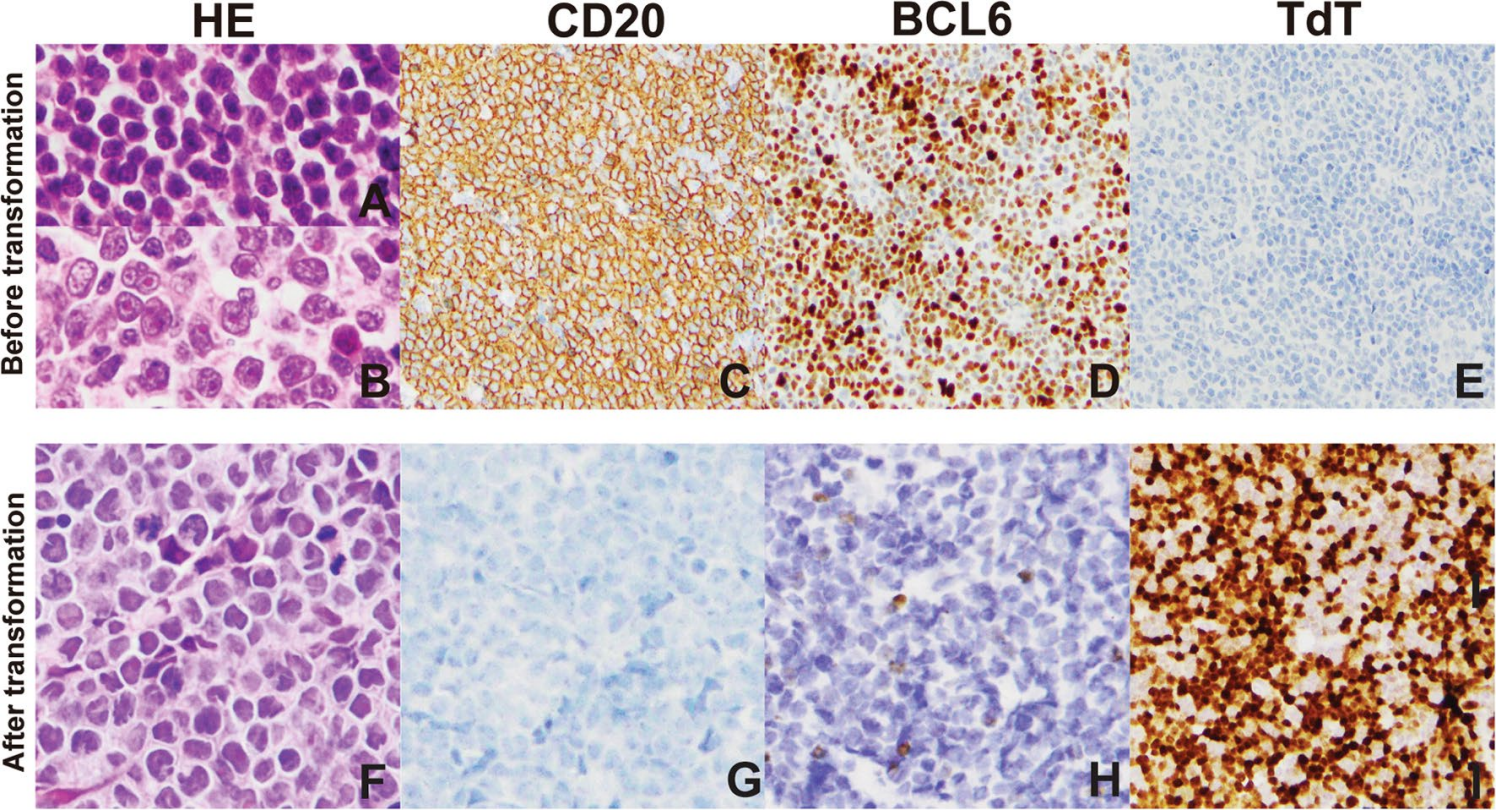
Representative pathological staining images. **A** HE staining showed that the lymph node structure was completely destroyed, with **B** significant hyperplasia of large cells with centroblastic and immunoblastic morphology in focal areas. IHC staining of **C** CD20, **D** BCL6, **E** TdT. **F** HE staining showed that the tumor cells presented with diffuse growth pattern. IHC staining of **G** CD20, **H** BCL6 and **I** TdT. (**A**–**E** represent the first biopsy examination, **F**–**I** represent the second biopsy examination)

**Fig. 3:**
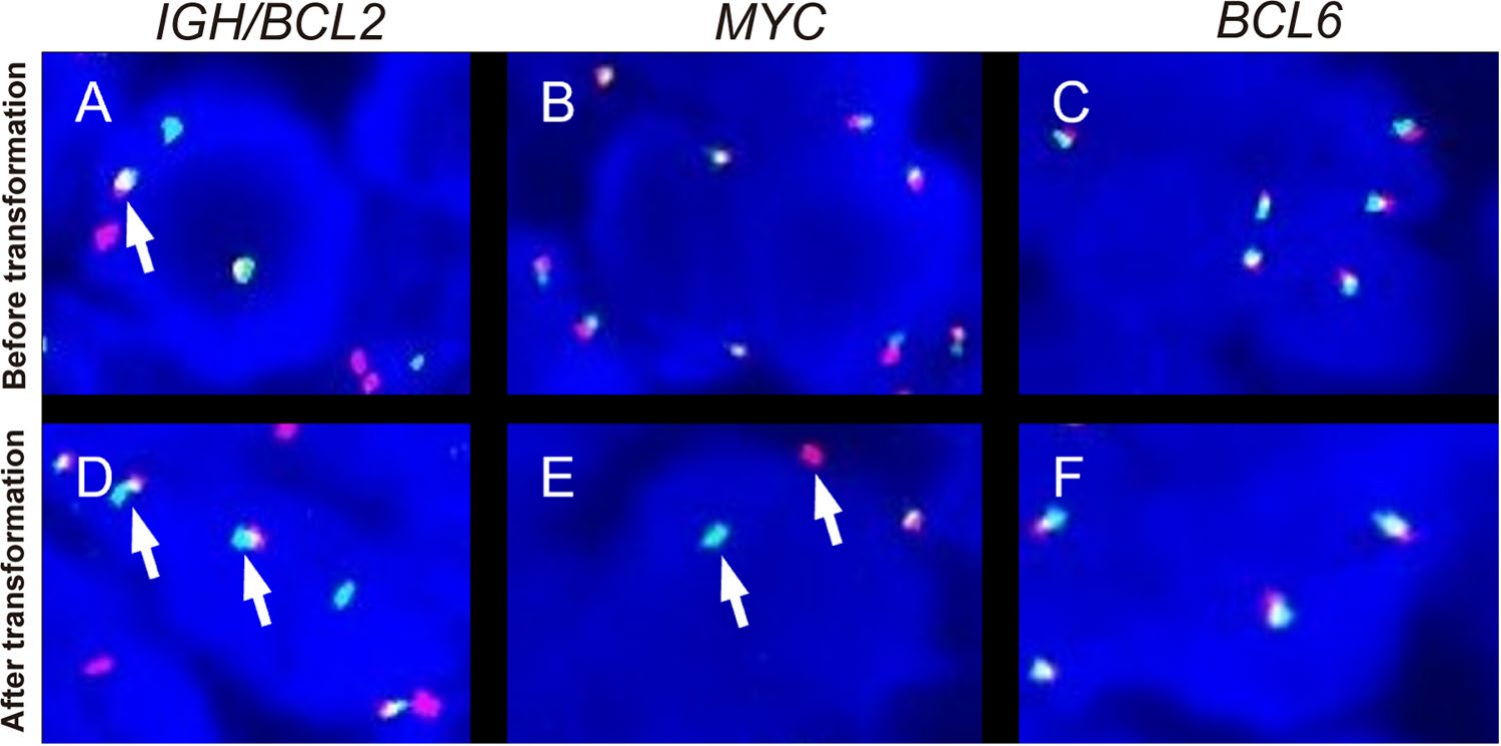
FISH analysis of primary (cervical lymph node) and transformed (abdominal mass) tissues. **A** Fusion signals indicating *IGH*/*BCL2* were seen in 71% interphase cells (*IGH*, green; *BCL2*, red). The fusion signals (yellow) are indicted by arrow. **B** Split signals for the *MYC* gene were seen in 8% (below threshold of 10%) interphase cells (*3’MYC*, green; *5’MYC*, red). **C** Split signals for the *BCL6* gene were seen in 2% (below threshold of 10%) interphase cells (*3’BCL6*, green; *5’BCL6*, red). **D** Fusion signals indicating *IGH*/*BCL2* were seen in 64% interphase cells. **E** Split signals for the *MYC* gene were seen in 45% interphase cells (arrows). **F** Split signals for the *BCL6* gene were seen in 1% interphase cells

In May 2018, a 43-year-old Chinese man was admitted to our hospital with lumbago and inguinal lymph node enlargement. Figure 1 summarizes the important events of this patient according to the timeline. The blood routine examination showed an increase in lactate dehydrogenase (LDH; 887 U/L) and a decrease in hemoglobin (Hb; 105 g/L). PET/CT showed multiple lymph nodes enlargements, including a large abdominal mass which was 13.9 × 10.6 cm in size, together with multiple bone lesions. The biopsy of cervical lymph node demonstrated that the lymph node structure was destroyed completely, which was mainly composed of small to medium-sized lymphocytes with round or slightly irregular nucleus, deeply stained chromatin, inconspicuous nucleoli, scant pale cytoplasm and rare apoptosis (Fig. 2A). Also, significant hyperplasia of large cells with centroblastic and immunoblastic morphology was seen in the focal areas, with large and round nucleus, condensed chromatin, one or more small nucleoli (Fig. 2B). Immunohistochemical (IHC) staining demonstrated the cells were positive for CD20 (Fig. 2C), CD19, CD10, BCL6 (Fig. 2D), BCL2 and negative for TdT (Fig. 2E), C-MYC, Cyclin D1, SOX11, CD23, LEF1, CD21, CD43, CD3, CD5 and EBER. Ki-67 was 10% in small cell areas and 40% in large cell areas. Fluorescence in situ hybridization (FISH) showed the cells were positive for *BCL2*::*IGH* fusion (Fig. 3A), without translocation of *MYC* or *BCL6* (Fig. 3B, C). Also, the biopsy of bone marrow was performed, and we observed the hyperplasia was extremely active with almost no adipose tissue, and the central mother large cells presented a diffuse growth pattern. The IHC result demonstrated that the cells were positive for CD19, CD20, CD79a, CD10, MUM1, Ki-67, BCL6, C-MYC and BCL2, while negative for CD3 and TdT. Thus, this patient was diagnosed with low grade FL (account for 80%) combined with DLBCL (20%) at a stage of IVB, and given R-CHOP regimen (rituximab, cyclophosphamide, doxorubicin, vindesine, prednisone). After 1 cycle of R-CHOP treatment, lumbago was significantly relieved and the treatment regimen was changed to HD-MTX (high dose methotrexate) combined with 80% dose R-EPOCH (rituximab, cyclophosphamide, doxorubicin, vindesine) due to the high IPI (international prognostic index) score of 4. The abdominal mass reduced to 7.5 × 3.5 and this patient achieved partial remission (PR) following 1 cycle of HD-MTX/R-EPOCH treatment. In the following 5 cycles of HD-MTX/R-EPOCH treatment, the abdominal mass was treated with additional 30 Gy radiotherapy. However, multiple masses in the abdominal cavity, many nodules around both kidneys and the right adrenal gland, were found after 6 cycles of the treatment, together with the increased glucose metabolism. Thus, he achieved progression disease (PD).

In Jan. 2019, a fine needle aspiration biopsy of the abdominal mass was taken. The biopsy demonstrated that the tumor cells presented with diffuse growth pattern, mainly composed of uniformly medium-sized blastoid cells with round or slightly irregular nuclei, dispersed chromatin, unclear nucleoli and scant bluish-grey cytoplasm (Fig. 2F). The cells were negative for CD20 (Fig. 2G), BCL6 (Fig. 2H) and CD3 staining, while positive for TdT (Fig. 2I), PAX5, CD38, CD10, BCL2, C-MYC and MUM1 staining. Ki-67 was about 60%. FISH revealed fusions of *BCL2*::*IGH* (Fig. 3D) and translocation of *MYC* (Fig. 3E, break apart probes), without *BCL6* translocation (Fig. 3F). According to the 5th WHO classification, a diagnosis of TdT positive HGBL with MYC and BCL2 rearrangements (“double hit”) transformed from FL/DLBCL was made after 8 months of FL/DLBCL confirmation. Then, chidamide and pembrolizumab were given and the disease was mitigated, but he discontinued chemotherapy for 3 months due to the COVID-19 pandemic, and the disease progressed. The biopsy of the abdominal neoplasm was taken once again in Apr. 2020, and the diagnosis of TdT positive “double hit” HGBL remained valid. This patient died in Jul. 2021, with an OS time of 37.8 months.

**Fig. 4:**
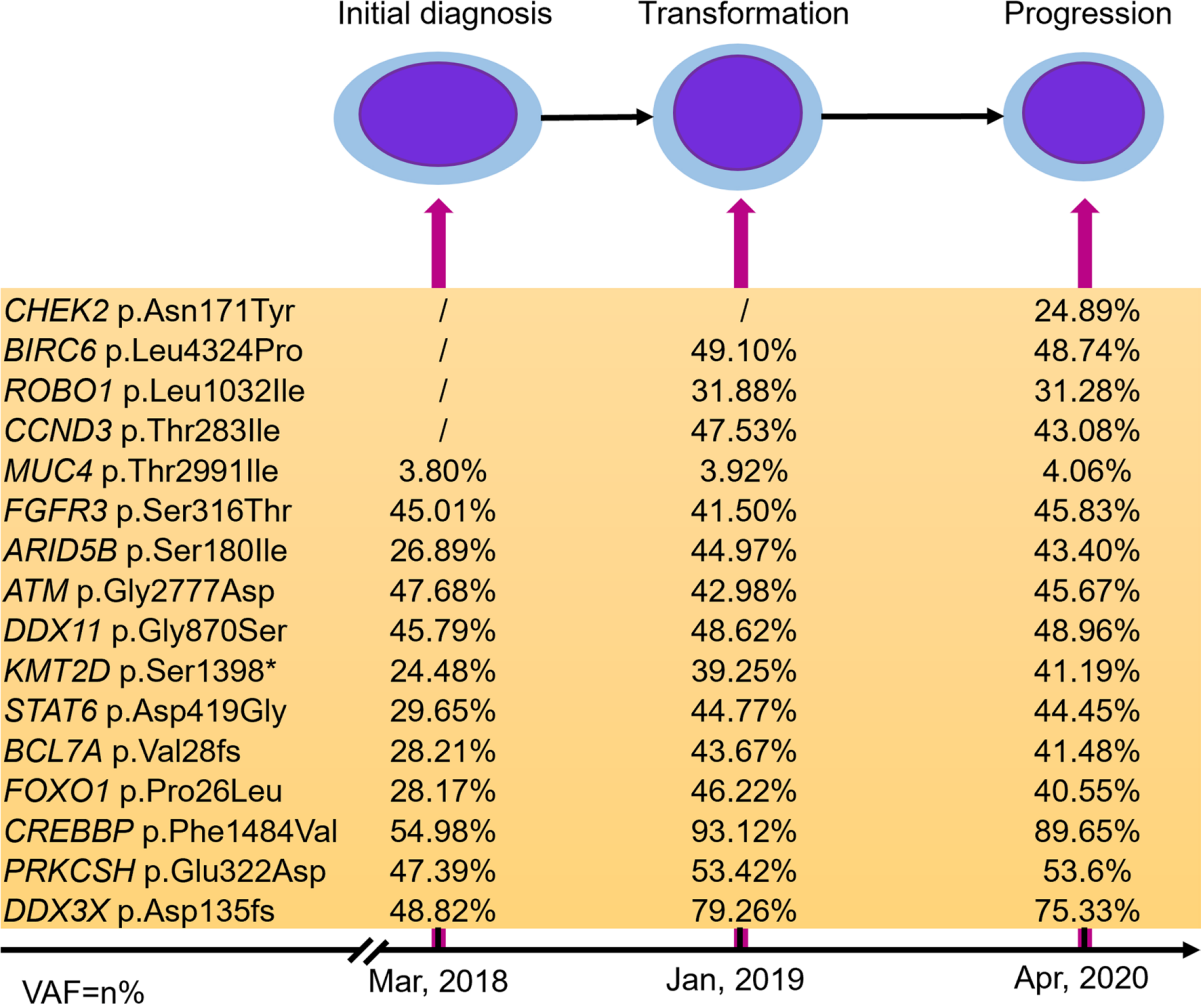
Patterns of genomic evolution. The yellow mutations at the bottom were the common mutations of the three sample, while transformed samples also acquired mutations in *CCND3*, *BIRC6*, *ROBO1* and *CHEK2* genes

To further understand the pathogenesis of TdT positive “double hit” HGBL after the treatment of FL/DLBCL, the targeted sequencing of 571 lymphoma-related genes (Supplementary Table 1) were carried out on Nova-seq (Illumina, San Diego, CA, USA). Variants, including SNVs (single nucleotide variations) and Indels (Insertion and deletion) were screened by Shanghai Rightongene Biotechnology Co., Ltd. (Shanghai, China) based on the following filter conditions: (1) SNVs or Indels with a mutation allele frequency (MAF) ≥ 0.001 in databases of 1000 genomes project [14], 1000 genome East Asian, ExAC all or ExAC East Asian and gnomAD [15] were removed; (2) SNVs or Indels with a variant allele frequency (VAF) ≥ 5% was retained; (3) SNVs or Indels including stopgain, stoploss, frameshift, non-frameshift and splicing sites were retained; and (4) Missense mutations with sift ≤ 0.05, Polyphen2_HVAR_pred ≥ 0.447 and CADD > 4 were retained. *KMT2D*, *FOXO1*, *CREBBP*, *ATM*, *STAT6*, *BCL7A*, *DDX3X*, *MUC4*, *FGFR3*, *ARID5B*, *DDX11* and *PRKCSH* were the co-mutations detected in both the primary and transformed tissues*,* while mutations in *CCND3*, *BIRC6*, *ROBO1* and *CHEK2* genes emerged when transformed into HGBL (Fig. 4).

## Discussion and conclusion

It is of crucial to distinguish mature B-cell lymphomas from immature or precursor B-cell neoplasms as different treatment regimens are given. Morphology provides important information, but it is well known that some mature B-cell neoplasms may demonstrate relatively immature morphologic features [13]. Furthermore, TdT expression in neoplastic cells have been considered as features to support immaturity [16–18]. However, growing evidence has demonstrated that TdT is also expressed in otherwise mature B-cell lymphomas, most often associated with *MYC* and *BCL2* and/or *BCL6* translocations, commonly transformed from FL and less often from chronic lymphocytic leukemia [19]. Since the underlying biology are essentially different from de novo lymphoblastic B-cell leukemia/lymphoma, these proliferations should not be classified as such and thus according to the 5th WHO classification, a diagnosis of TdT positive HGBL with *MYC* and *BCL2* rearrangements transformed from the indolent lymphoma, from which it has evolved, should be made. In this study, we reported a case diagnosed with HGBL with *MYC* and *BCL2* rearrangements after 8 months of FL/DLBCL with the expression of TdT. Regrettably, we cannot conclude that whether the HGBL occurred at the same time or after the FL/DLBCL as we did not perform pathological examination on the abdominal mass at initial diagnosis. The following evidence can support the transformation of TdT positive HGBL from FL/DLBCL, first, the size of abdominal mass was significantly reduced from 13.9 × 10.6 cm to 7.5 × 3.5 cm after the treatment; second, a small number of TdT positive cells were scattered in the focal area the of biopsy of chest wall; third, BCL/IGH rearrangement, which often occurred in FL, was detected in both the primary and secondary biopsies; forth, *CREBBP* and *KMT2D* mutations, which were common in FL [20, 21], were ever persist in primary biopsy of FL and the secondary biopsy of HGBL.

However, some cases similar with ours were reported as B-lymphoblastic lymphoma/leukemia in the literature according to the 4th revised WHO classification, and a total of 28 similar cases were reported previously [17, 22–35]. Generally, the histologic transformation period of FL to other aggressive lymphoma, usually DLBCL, ranges from 10 to 15 years from the initial diagnosis [36], but it declined when lymphoblastic transformation occurred [31]. The interval time in our patient was 8 months, which was within the transformed period from FL to B-lymphoblastic lymphoma/leukemia (from 2.75 to 72 months), as Nie et al. [17] reported. The median OS for patients with other aggressive lymphoma transformation from FL is about 50 months, and the most common treatment is R-CHOP-based therapy [37]. However, most patients with lymphoblastic transformation survive for less than one year after diagnosis after transformation of FL [31, 37], which may be explained by the evolution from dedifferentiation of lymphoma cells. Different regimens such as CHOP-based methods are given to these patients depending on patients’ conditions [31, 33]. This patient survived 17.8 months following the diagnosis of HGBL. Compared with other cases, the co-occurrence of DLBCL content with FL at the initial diagnosis, as well as the treatment regimen (pembrolizumab combined with chidamide) may cause this difference. Pembrolizumab is one of the two PD-1 inhibitors approved to for the treatment of relapsed and refractory classical Hodgkin lymphoma (cHL) and primary mediastinal large B cell lymphoma (PMBL) to date. However, immune checkpoint blockade monotherapy did not prove to be effective either aside from cHL and PMBL [38]. In addition, studies have found that the combination of PD-1 inhibitor and HDAC inhibitor chidamide is effective in immunotherapy-resistant NK/T-cell lymphoma [39] and B cell lymphoma [40]. To our surprise, he relieved following this regimen and the remission status maintained for a long time. We conjecture that the combination of pembrolizumab and chidamide may be a influencing factor to prolong the survival of TdT positive “double hit” HGBL transformed from FL/DLBCL, as demonstrated by this case.

*BCL2* gene rearrangement is a common feature of FL/DLBCL, as well as the preexisting FL cases with HGBL soon afterwards [11, 18]. For instance, Geyer et al. [31] found *BCL2* gene rearrangement was detected in 4 of 5 cases with lymphoblastic transformation of FL. Consistently, this case also presented with *IGH::BCL2* translocation at the initial diagnosis of FL/DLBCL and the following diagnosis of TdT positive “double hit” HGBL. In addition, almost all reported FL cases with lymphoblastic transformation have *MYC* gene rearrangement, while *MYC* rearrangement is detected only in 20% of preexisting FLs [17, 30, 33, 41]. Conformably, *MYC* rearrangement was not detected in the initial FL/DLBCL sample, but occurred following the transformation. In addition, the FL cases transforming to HGBL are always at low grade except one case who was transformed from FL grade 3A reported recently [17].

Efforts at molecular level are also being made to reveal the pathogenesis of lymphoblastic transformation of FL. Nie et al. [17] described the mutation landscapes of B-lymphoblastic transformation of FL in 4 cases using the whole-exon sequencing (WES). Common mutations such as the mutations of *CREBBP* were shared by the FL and B-lymphoblastic lymphoma [17]. Herein, mutations in *KMT2D*, *FOXO1*, *CREBBP*, *ATM*, *STAT6*, *BCL7A*, *DDX3X*, *MUC4*, *FGFR3*, *ARID5B*, *DDX11* and *PRKCSH* genes were ever-present in both primary and transformed tissues. According to the simplified algorithm for genetic subtyping in DLBCL published by Shen et al. [42], this patient at initial diagnosis of FL/DLBCL was assigned to the EZB-like subtype. This subtype is characterized with *BCL2* fusion together with mutations in *EZH2*, *TNFRSF14*, *KMT2D*, B2M, *FAS*, *CREBBP*, *ARID1A*, *EP300*, *CIITA*, *STAT6*, and *GNA13* genes. Generally, the prognosis for this subtype is better than other subtypes especially for MCD-like, TP53^mut^ and N1, with a 5-year survival rate of 60–80% [42]. This patient survived 37.8 months after the diagnosis of FL/DLBCL, and HGBL transformation may cause this decreased survival. *CREBBP*, a tumor-suppressor gene, is frequently mutated in FL and DLBCL, and permanently mutated during the transformation to HGBL [17], suggesting a prerequisite role of *CREBBP* mutations in HGBL transformation of FL. In addition, we found *KMT2D* mutation was also detected in both FL and transformation samples, which was consistent with the study of Slot et al. [33]. *KMT2D* is a frequent mutated gene in FL and is identified as a driver gene of FL [20]. Mutations in *BIRC6*, *ROBO1*, *CCND3* and *CHEK2* genes were uniquely present following the treatment of initial diagnosis. *BIRC6* is the largest member of the IAP (inhibitors of apoptosis protein) family which triggers apoptosis resistance [43], the mutation of which is common in gray zone lymphomas [44]. *ROBO1*, a member of the Roundabout family, was originally considered to regulate axon growth and control the central nervous system midline crossing [5], but now has been described as a tumor suppressor gene, whose mutation has been discovered in B-cell malignancies [45]. Cyclin D3 encoded by *CCND3* plays a vital role in germinal center B cell proliferation, especially in the dark zone, and the mutation of *CCND3* is detected in Burkitt lymphoma [46, 47]. Consistently, Slot et al. [33] reported that *CCND3* mutation was also uniquely detected in the precursor B-lymphoblastic lymphoma. *CHEK2* (checkpoint kinase 2) coded protein is an important mediator of the DNA damage response pathway, and *CHEK2* mutation is reported to be associated with an unfavorable prognosis in non-Hodgkin lymphoma (NHL) [47], suggesting the worse prognosis of this case may be also related to the *CHEK2* mutation. No direct evidence demonstrates the B-lymphoblastic transformation-related mutation, and we conjecture mutations in *CREBBP, KMT2D, BIRC6*, *ROBO1*, *CCND3* and *CHEK2* may involve the HGBL of FL, which should be verified in larger samples.

Overall, the present study reported a case with HGBL transformation from FL/DLBCL. The common features of this kind of cases include TdT expression, *BCL* and *MYC* rearrangement, *CREBBP* and *KMT2D* mutations, rapid progression and poor outcome.

### Supplementary Information

Additional file1 (XLSX 15 KB)

## Data Availability

Data is provided within the manuscript or Additional files; further inquiries can be directed to the corresponding authors.

## Abbreviations

*CHEK2*: Checkpoint kinase 2
DLBCL: Diffuse large B-cell lymphoma
FISH: Fluorescence in situ hybridization
FL: Follicular lymphoma
Hb: Hemoglobin
HD-MTX: High dose methotrexate
HGBL: High grade B cell lymphoma
IAP: Inhibitors of apoptosis protein
IHC: Immunohistochemical
Indels: Insertion and deletion
IPI: International prognostic index
LDH: Lactate dehydrogenase
MAF: Mutation allele frequency
NHL: Non-Hodgkin lymphoma
OS: Overall survival
PD: Progression disease
PR: Partial remission
R-CHOP: Rituximab, Cyclophosphamide, Doxorubicin, Vindesine, Prednisone
R-EPOCH: Rituximab, Cyclophosphamide, Doxorubicin, Vindesine
SNVs: Single nucleotide variations
TdT: Terminal deoxynucleotidyl transferase
VAF: Variant allele frequency
WES: Whole-exon sequencing
